# Helminth Infections Coincident with Active Pulmonary Tuberculosis Inhibit Mono- and Multifunctional CD4^+^ and CD8^+^ T Cell Responses in a Process Dependent on IL-10

**DOI:** 10.1371/journal.ppat.1004375

**Published:** 2014-09-11

**Authors:** Parakkal Jovvian George, Rajamanickam Anuradha, Nathella Pavan Kumar, Rathinam Sridhar, Vaithilingam V. Banurekha, Thomas B. Nutman, Subash Babu

**Affiliations:** 1 ICER Department, National Institutes of Health—NIRT—International Center for Excellence in Research, Chennai, India; 2 Department of Thoracic Medicine Government Stanley Medical Hospital, Chennai, India; 3 Department of Clinical Research, National Institute for Research in Tuberculosis, Chennai, India; 4 Laboratory of Parasitic Diseases, National Institutes of Allergy and Infectious Diseases, National Institutes of Health, Bethesda, Maryland, United States of America; University of Medicine & Dentistry New Jersey, United States of America

## Abstract

Tissue invasive helminth infections and tuberculosis (TB) are co-endemic in many parts of the world and can trigger immune responses that might antagonize each other. We have previously shown that helminth infections modulate the Th1 and Th17 responses to mycobacterial-antigens in latent TB. To determine whether helminth infections modulate antigen-specific and non-specific immune responses in active pulmonary TB, we examined CD4^+^ and CD8^+^ T cell responses as well as the systemic (plasma) cytokine levels in individuals with pulmonary TB with or without two distinct helminth infections—*Wuchereria bancrofti* and *Strongyloides stercoralis* infection. By analyzing the frequencies of Th1 and Th17 CD4^+^ and CD8^+^ T cells and their component subsets (including multifunctional cells), we report a significant diminution in the mycobacterial–specific frequencies of mono- and multi–functional CD4^+^ Th1 and (to a lesser extent) Th17 cells when concomitant filarial or Strongyloides infection occurs. The impairment in CD4^+^ and CD8^+^ T cell cytokine responses was antigen-specific as polyclonal activated T cell frequencies were equivalent irrespective of helminth infection status. This diminution in T cell responses was also reflected in diminished circulating levels of Th1 (IFN-γ, TNF-α and IL-2)- and Th17 (IL-17A and IL-17F)-associated cytokines. Finally, we demonstrate that for the filarial co-infections at least, this diminished frequency of multifunctional CD4^+^ T cell responses was partially dependent on IL-10 as IL-10 blockade significantly increased the frequencies of CD4^+^ Th1 cells. Thus, co-existent helminth infection is associated with an IL-10 mediated (for filarial infection) profound inhibition of antigen-specific CD4^+^ T cell responses as well as protective systemic cytokine responses in active pulmonary TB.

## Author Summary

While it has long been recognized that helminth infections alter the pathophysiology of allergic and autoimmune disease, data suggest that helminth infections also exert an important immunological effect on concomitant infections and vaccine responses. In particular, helminth coinfection can modulate the severity, pathogenesis and transmission of other infectious diseases. In this study, we examine the mechanism by which helminth infections modulate the immunological responses to tuberculosis antigens in individuals with active pulmonary tuberculosis. Our data suggest that two different helminth infections, with different life cycles, tissue localization and modes of transmission essentially exert very similar effects on the adaptive immune response to tuberculosis antigens in pulmonary tuberculosis. This includes a compromised induction of protective cytokine-expressing T cells as well as inhibitory effects on systemic cytokines that are potentially protective in tuberculosis. The strength of this study lies in the fact that this is the first study to demonstrate that two different helminth infections essentially impair cytokine responses in a similar manner in pulmonary tuberculosis.

## Introduction

Helminth parasites are complex eukaryotic organisms, characterized by their ability to maintain long-standing infections in humans, sometimes lasting decades. Two of the most common persistent helminth infections are *Wuchereria bancrofti*, the major causative agent of lymphatic filariasis, and *Strongyloides stercoralis*, the causative agent of strongyloidiasis together infecting close to 250 million people worldwide [1], [2]. In addition, both these infections are often clinically asymptomatic due, in large part, to the parasites' ability to manipulate the host immune system, a feature that insures their survival largely because of their ability to restrict local inflammatory pathology [3], [4]. Modulation of the host immune response involves a variety of strategies including the induction of regulatory networks that leads to dysregulation of innate and adaptive immune responses [3], [4]. The immune down modulation associated with helminth infections is primarily parasite-antigen specific, but some bystander effects on parenterally administered vaccine responses, allergen skin test positivity, non-helminth pathogen-specific immune responses and autoimmune diseases have been noted [5], [6], [7]. In terms of interaction in human TB, filarial infections have been shown to alter the antigen - specific protective immune responses in latent TB by modulating the Th1 and Th17 responses to TB antigens [8]. In addition, Strongyloides has been shown to alter the protective Th17 cytokine responses in animal models of co-infection [9]. Finally, helminth infections are strongly associated with an IL-10 dominant regulatory environment that could potentially down modulate antigen - specific responses to third party antigens [10].

Active TB reflects the progression from latent TB to active symptomatic disease that is usually attributed to failure to contain Mtb within a granuloma. However, it is well established that the control of TB infection is dependent on Th1 (IL-12, IFN-γ and TNF-α) and, to a lesser extent, Th17 (IL-17 and IL-23) responses [11]. Both Th1 and Th17 responses have been shown to be important in the induction and maintenance of protective immune responses in mouse models of TB infection or for control of human TB infection (as seen in latent TB) [12], [13], [14]. The presence of multifunctional T cells (expressing more than one cytokine) has also been shown to be an important correlate of protective immunity to a wide variety of pathogens, including TB [15]. Multifunctional CD4^+^ Th1 cells, co-expressing IFN-γ/TNF-α/IL-2 or IFN-γ/IL-2 or IFN-γ/TNF-α have been shown to be associated with protection against active pulmonary disease in TB [16], [17], [18]. In addition, the absence or reduced frequency of multifunctional Th1 cells is thought to correlate with the severity of TB disease [19]. Th17 cells have also been shown to play a role in protection against TB infection as well as in the induction of memory responses in animal models [12]. However, the role of multifunctional Th17 cells, if any, in active human pulmonary TB remains unexplored. Finally, Type 1 and Type 17 cytokine production by CD8^+^ T cells is also thought to play an important role in protection against TB infection/disease [12].

Helminth infections commonly occur throughout the tropics and subtropics and in many regions of the world have an overlapping geographic distribution with *Mycobacterium tuberculosis* (Mtb) [6]. Moreover, age-specific prevalence studies have indicated that helminth infections usually precede the acquisition of pulmonary tuberculosis [20]. Finally, both filarial parasites (present in the circulation) and Stronglyoides (which is an intestinal helminth but has a lung migratory larval stage) could directly influence the outcome of TB infection. We therefore hypothesized that immune responses in active TB might be modulated by the regulatory immune networks often seen in chronic helminth infections that could have a negatively impact on the course of active TB. To this end, we examined CD4^+^ and CD8^+^ Th1 and Th17 responses in patients with active TB with or without concomitant filarial or Stronglyloides infection. Our data suggest that coincidental helminth infection has a profound inhibitory effect on multi - functional Th1 and Th17 responses as well as on systemic cytokine responses in active pulmonary TB. Our data also suggest that IL-10 is an important mediator of these inhibitory effects for filarial co-infections.

## Results

### Coincident helminth infection is not associated with altered CD4^+^ and CD8^+^ T cell counts or subset frequencies in active pulmonary tuberculosis

To determine the impact of helminth infection on the hematological and immunological parameters of active TB individuals at baseline (or steady state), we performed hematological and flow cytometry analysis on these individuals. As shown in Table 1, infection with *W. bancrofti* or *S. stercoralis* in the context of active pulmonary TB was not associated with significant alterations in the absolute numbers of CD4^+^ and CD8^+^ T cells nor in the frequency distribution of the various T cell subsets - naive, central memory, effector memory and regulatory T cells - when compared to helminth-uninfected individuals with active TB. Similarly, all other hematological and immunological parameters examined including total leukocyte and differential cell counts were similar between those helminth-infected and –uninfected individuals with active TB.

**Table 1:** Demographic, hematology and immunological profiles.

	FIL/TB	STR/TB	TB	p value
	n = 17	n = 13	n = 20	
Age	45 (19–65)	54 (19–65)	51 (23–70)	NA
Sex: M/F	16/1	12/1	16/4	NA
Smear Grade: 0/1+/2+/3+	4/8/0/5	0/2/8/3	2/8/4/6	NA
Circulating Filarial Antigen (CFA) level	1192.54 (212–5998) IU/mL	<32 IU/mL	<32 IU/mL	NA
Circulating recombinant Strongyloides antigen (NIE) level	<10212 LU/mL	304463.231 (1127–1985444) LU/mL	<10212 LU/mL	NA
RBC 10^6^/mL	4.43 (2.93–5.73)	4.8 (3.56–8.03)	4.78 (3.76–5.79)	NS
WBC 10^3^/mL	12.14 (7.1–21.2)	11.52 (7.2–19.1)	10.37 (6.9–15.4)	NS
Hgb g/dL	12 (8.3–17.3)	14.48 (10–22.8)	12.77 (7.7–16.7)	NS
HCT %	34.47 (22.1–46.5)	36.54 (26.94–45)	37.46 (23.2–51.5)	NS
PLT 10^3^/mL	415.12 (305–640)	342.72 (175–652)	349.59 (146–674)	NS
Neutrophils 10^3^/mL	8.64 (4.8–16.05)	8.05 (4.3–16.05)	6.89 (4.44–12.58)	NS
Lymphocytes 10^3^/mL	1.67 (0.94–3.3)	1.75 (0.94–3.31)	2.03 (1.15–3.53)	NS
Monocytes 10^3^/mL	1.09 (0.58–1.9)	0.87 (0.46–1.33)	0.75 (0.24–1.42)	NS
Eosinophils 10^3^/mL	0.3 (0.07–1.44)	0.44 (0.22–0.8)	0.32 (0.07–1.98)	NS
Basophils 10^3^/mL	0.08 (0.01–0.22)	0.08 (0.03–0.18)	0.07 (0.03–0.18)	NS
CD4+T cells (absolute number)	729.63 (291–1603)	615.37 (226–1284)	771.07 (226–2986)	NS
Effector memory CD4+Tcells (%)	23.6 (8.7–61.4)	34.06 (20–61.4)	18.86 (2.12–53.3)	NS
Central memory CD4+Tcells (%)	27.33 (5.08–53.2)	26.19 (10.65–43.2)	28.06 (8.87–57.9)	NS
Naïve CD4+Tcells (%)	30.79 (15.2–49.9)	33.8 (15.12–58.3)	36.51 (9.16–79.6)	NS
CD4+ regulatory T cells (%)	4.23 (0.825–8.859)	4.2 (2.35–7.17)	4.63 (6.629–12.129)	NS
CD8+T cells (absolute number)	514.5 (238–1507)	394.74 (240–658)	557.79 (152–3842)	NS
Effector memory CD8+Tcells (%)	27.1 (14.1–53.8)	23.71 (23.2–49.8)	23 (1.33–55.5)	NS
Central memory CD8+Tcells (%)	20.92 (5.28–68.8)	21.16 (3.71–62.8)	20.35 (2.63–50)	NS
Naïve CD8+Tcells (%)	2.53 (0.324–20.8)	2.85 (0.77–30)	1.75 (0.137–33)	NS
Classical monocytes (%)	6.79 (4.11–12.1)	7.11 (5.7–9.365)	6.58 (3.23–10.00)	NS
Intermediate monocytes (%)	1.29 (0.216–4.19)	0.88 (0.139–3.985)	0.98 (0.286–4.06)	NS
Non-classical monocytes (%)	0.59 (0.077–3.82)	0.342 (0.189–0.685)	0.53 (0.111–2.43)	NS

### Coincident helminth infection is associated with decreased frequencies of mycobacterial-antigen specific mono- and multi- functional CD4^+^ Th1 cells

**Figure 1:**
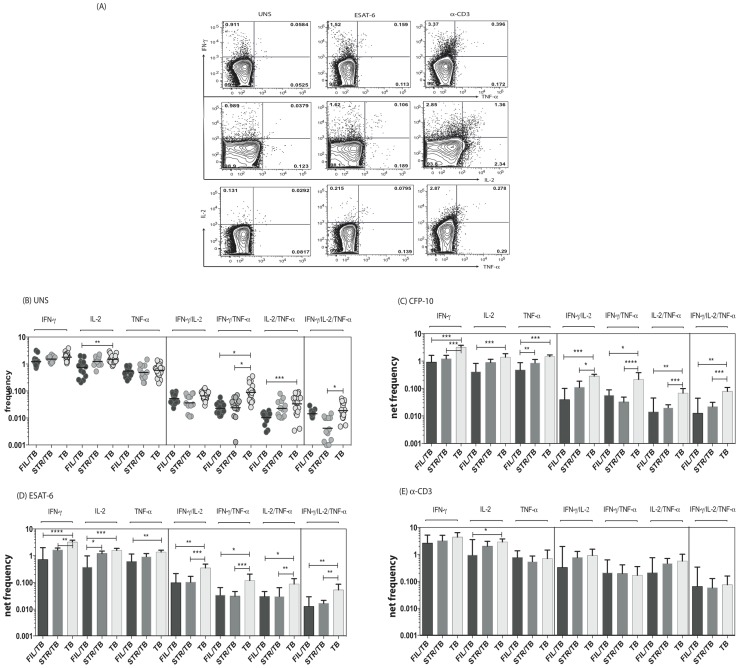
Helminth infections are associated with decreased frequencies of mono- and multifunctional mycobacterial antigen-specific CD4^+^ Th1 cells in active TB. Whole blood was cultured with media alone, CFP-10, ESAT-6 or anti-CD3 for 6 h and the baseline or net frequencies of mono - and multifunctional Th1 cells determined. (A) Representative whole blood intracellular cytokine assay flow data from an active TB individual showing expression of Th1-associtated cytokines. The plots shown are gated on CD3^+^CD4^+^ T cells. (B-E) The spontaneous frequencies (B) are shown as scatter plots the net frequencies of mono– and multifunctional CD4^+^ Th1 cells following CFP-10 (C) or ESAT-6 (D) or anti-CD3 (E) stimulation are shown as bar graphs. All individuals had pulmonary TB with concomitant filarial infection (FIL/TB, n = 17) or concomitant Strongyloides infection (STR/TB, n = 13) or no helminth infection (TB, n = 20). The bars represent the geometric mean and 95% confidence intervals. Net frequencies were calculated by subtracting baseline frequency from the antigen–induced or anti-CD3 induced frequency for each individual. P values were calculated using the Kruskal-Wallis test with Dunn's multiple comparisons (* p<0.05, ** p<0.01, *** p<0.001).

Since a decrease in multifunctional CD4^+^ Th1 cells is known to be associated with increased bacterial burdens in active TB [19] and since both mono - and multifunctional CD4^+^ Th1 cells are potential correlates of protective immunity in TB [11], we sought to determine the impact of helminth infection on both the mono-functional and multifunctional CD4^+^ Th1 responses in TB infected individuals. To this end, we cultured whole blood from FIL/TB, STR/TB and TB only individuals with media alone, CFP-10, ESAT-6 and anti-CD3 and measured the frequency of CD4^+^ T cells expressing each of the Th1-associated cytokines (Figure 1A). As shown in Figure 1B, co-incidental filarial infection was associated with significantly lower frequencies of CD4^+^ T cells expressing IL-2 alone or co – expressing TNF-α/IFN-γ or IL-2/IFN-γ at baseline. Similarly, Strongyloides co-infection was associated with decreased frequencies of CD4^+^ T cells co-expressing TNF-α/IFN-γ or IL-2/IFN-γ/TNF-α at baseline in comparison to individuals with active TB only. In addition, as shown in Figures 1C and D, co-incidental filarial infection was associated with significantly lower frequencies of CFP-10 and ESAT-6 induced net frequencies of CD4^+^ T cells expressing IL-2 or IFN-γ or TNF-α alone or co-expressing TNF-α/IFN-γ or IL-2/IFN-γ or IL-2/TNF-α or IL-2/IFN-γ/TNF-α in comparison to individuals with active TB only. Similarly, Stronglyloides co-infection was also associated with significantly decreased frequencies of almost all of the above-mentioned mono - and multifunctional CD4^+^ Th1 cell subsets in response to CFP-10 and ESAT-6 (Figures 1C and D). Finally, no significant differences in the net frequency of CD4^+^ Th1 cells was observed between the helminth-infected and -uninfected groups following anti-CD3 stimulation, with the exception of CD4^+^ T cells expressing IL-2 alone in FIL/TB individuals (Figure 1E). Thus, helminth infections are associated with a down modulation of spontaneous and/or antigen - specific mono - and multifunctional Th1 responses in active TB.

### Coincident helminth infection is associated with decreased frequencies of mycobacterial-antigen specific mono- and multifunctional CD4^+^ Th17 cells

**Figure 2:**
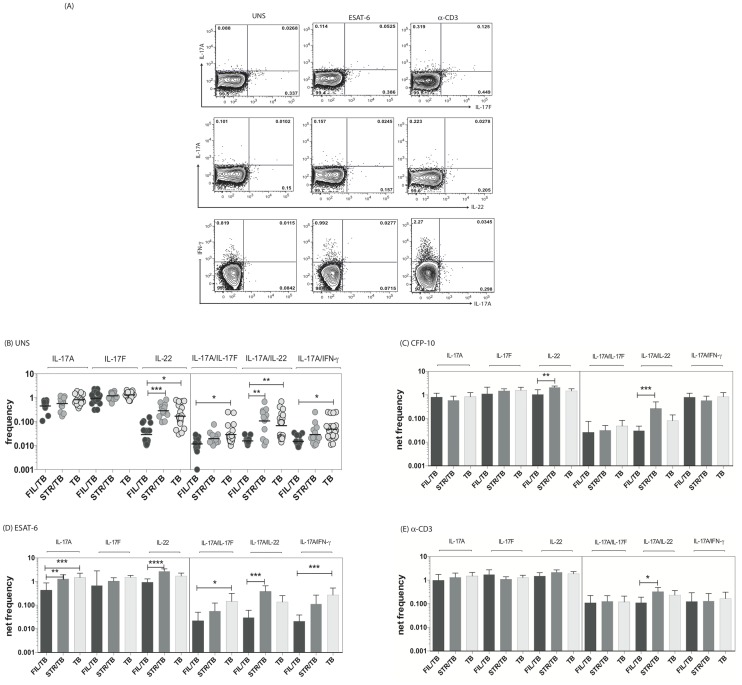
Helminth infections are associated with decreased frequencies of mono- and multifunctional mycobacterial antigen-specific CD4^+^ Th17 cells in active TB. Whole blood was cultured with media alone, CFP-10, ESAT-6 or anti-CD3 for 6 h and the baseline or net frequencies of mono - and multifunctional Th17 cells determined. (A) Representative whole-blood intracellular cytokine assay flow data from an active TB individual showing expression of Th17 cytokines. The plots shown are gated on CD3^+^CD4^+^ T cells. (B–E) The spontaneous frequencies (B) are shown as scatter plots and the net frequencies of mono– and multifunctional CD4^+^ Th17 cells following CFP-10 (C) or ESAT-6 (D) or anti-CD3 (E) stimulation are shown as bar graphs. All individuals had pulmonary TB with concomitant filarial infection (FIL/TB, n = 17) or concomitant Strongyloides infection (STR/TB, n = 13) or no helminth infection (TB, n = 20). The bars represent the geometric mean and 95% confidence intervals. Net frequencies were calculated by subtracting baseline frequency from the antigen–induced or anti-CD3 induced frequency for each individual. P values were calculated using the Kruskal-Wallis test with Dunn's multiple comparisons (* p<0.05, ** p<0.01, *** p<0.001).

Since both mono - and multifunctional CD4^+^ Th17 cells have also been implicated as being important in the immune response in active TB [11], we sought to determine the impact of helminth infection on the CD4^+^ Th17 responses in TB infected individuals. To this end, we cultured whole blood from co-infected (FIL/TB or STR/TB) and TB only (TB) individuals with media alone, CFP-10, ESAT-6 and anti-CD3 and measured the frequency of CD4^+^ T cells expressing each of the Th17-associated cytokines (Figure 2A). As shown in Figure 2B, at baseline, the frequencies of CD4^+^ T cells expressing IL-22 or co – expressing IL-17A/IFN-γ or IL-17A/IL-17F or IL-17A/IL-22 was significantly reduced in FIL/TB compared to TB alone individuals. In addition, as shown in Figures 2C and D, upon Mtb-specific antigen stimulation, the frequencies of CD4^+^ T cells expressing IL-17A or IL-22 or co – expressing IL-17A/IFN-γ or IL-17A/IL-17F was significantly reduced in FIL/TB compared to TB alone individuals. Moreover, similar to the pattern observed in Th1 cells, the differential frequencies of Th17 cells was also mycobacterial - antigen specific since anti-CD3 stimulated frequencies of these cells did not exhibit any major significant differences (Fig. 2E). In contrast, STR/TB individuals did not exhibit any significant differences in the frequencies of mono - or multifunctional CD4^+^ Th17 cells in comparison to TB alone individuals ex vivo or following stimulation with TB antigens or anti-CD3. Thus, helminth infections, and more specifically filarial infections, are associated with a modulation of spontaneous or antigen - specific Th17 responses in active TB.

### Coincident helminth infection is associated with decreased frequencies of mycobacterial-antigen specific Th1 and Th17 cytokine expressing CD8^+^ T cells

**Figure 3:**
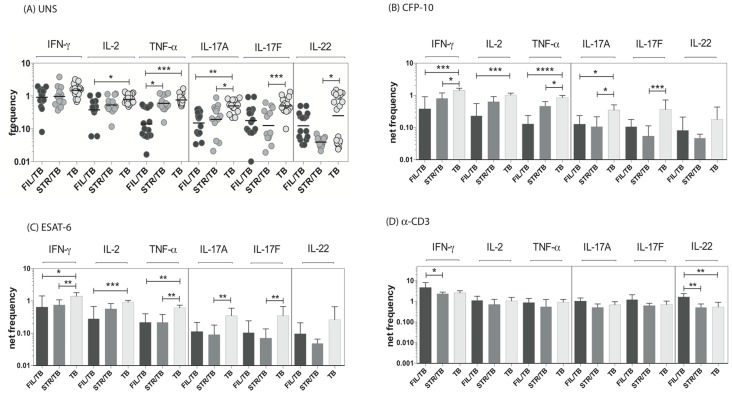
Helminth infections are associated with decreased frequencies of mycobacterial antigen-specific CD8^+^ Th1 and Th17 cells in active TB. Whole blood was cultured with media alone, CFP-10, ESAT-6 or anti-CD3 for 6 h and the baseline or net frequencies of mono - and multifunctional Th17 cells determined. The spontaneous frequencies with media alone (A) are shown as scatter plots and the net frequencies of CD4^+^ Th1 and Th17 cells following CFP-10 (B) or ESAT-6 (C) or anti-CD3 (D) stimulation are shown as bar graphs. All individuals had pulmonary TB with concomitant filarial infection (FIL/TB, n = 17) or concomitant Strongyloides infection (STR/TB, n = 13) or no helminth infection (TB, n = 20). The bars represent the geometric mean and 95% confidence intervals. Net frequencies were calculated by subtracting baseline frequency from the antigen – induced or anti-CD3 induced frequency for each individual. P values were calculated using the Kruskal-Wallis test with Dunn's multiple comparisons (* p<0.05, ** p<0.01, *** p<0.001).

Since CD8^+^ T cells play an important role in protection against TB [21], we sought to determine the impact of helminth infection on the CD8^+^ Th1 and Th17 cytokine responses in TB infected individuals. To this end, we cultured whole blood from co-infected (FIL/TB or STR/TB) and TB only individuals with media alone, CFP-10, ESAT-6 and anti-CD3 and measured the frequency of CD8^+^ T cells expressing each of the Th1 and Th17-associated cytokines. As shown in Fig. 3A, FIL/TB individuals exhibited significantly lower frequencies of CD8^+^ T cells expressing IL-2 or TNF-α or IL-17A in comparison to TB alone individuals ex vivo. Similarly, STR/TB individuals also exhibited significantly decreased frequencies of CD8^+^ T cells expressing IL-17A or IL-17 or IL-22 ex vivo (Figure 3A). In addition, FIL/TB individuals exhibited significantly lower frequencies of CD8^+^ T cells expressing IL-2 or INF-γ or TNF-α or IL-17A or IL-17F in comparison to TB only individuals upon CFP-10 and ESAT-6 stimulation (Figures 3B and C). Similarly, as shown in Figures 3B and C, STR/TB individuals also exhibited significantly lower frequencies of CD8^+^ T cells expressing INF-γ or TNF-α or IL-17A or IL-17F in comparison to TB only individuals upon CFP-10 and ESAT-6 stimulation. In contrast, the frequencies of CD8^+^ T cells expressing Th1 and Th17 cytokines was not significantly different between the 3 groups upon anti-CD3 stimulation. Thus, helminth infections are also associated with a down modulation of CD8^+^ T cell responses in active TB.

### Coincident helminth infection is associated with reduced circulating levels of Th1- and Th17-related cytokines in active pulmonary tuberculosis

**Figure 4:**
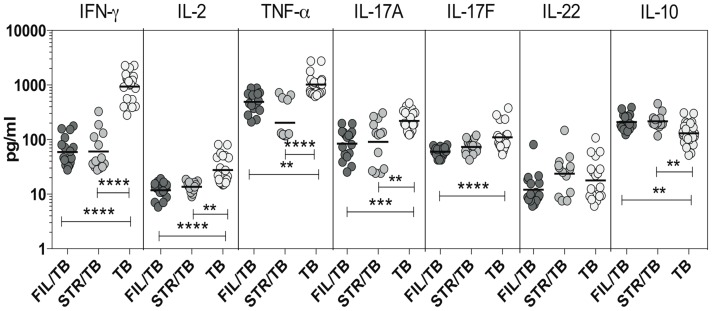
Helminth infections are associated with diminished plasma levels of Th1 and Th17 cytokines in active TB. The plasma levels of Th1- IFN-γ, IL-2, TNF-α and Th17– IL-17A, IL-17F, IL-22 cytokines as well as IL-10 were measured by ELISA in active pulmonary TB individuals with concomitant filarial infection (FIL/TB, n = 17) or concomitant Strongyloides infection (STR/TB, n = 13) or no helminth infection (TB, n = 20). The results are shown as scatterplots with each circle representing a single individual. P values were calculated using the Kruskal-Wallis test with Dunn's multiple comparisons (* p<0.05, ** p<0.01, *** p<0.001, **** p<0.0001).

Since Th1 and Th17 cytokines are cytokines important components of the immune response in active TB [11], we wanted to explore the effect of coincident helminth infection on systemic levels of these cytokines. To determine the impact of helminth infections on the circulating levels of the prototypical Th1 and Th17 cytokines as well as regulatory cytokines, we measured the levels of IFN-γ, TNF-α, IL-2, IL-17A, IL-17F,IL-22, IL-10 and TGFβ in the plasma of three groups of individuals with active TB - FIL/TB, STR/TB or TB alone. As shown in Figure 4, we observed significantly lower plasma levels of Th1 associated cytokines - IFN-γ (Geometric Mean of 936.6 pg/ml in TB alone vs. 59.2 pg/ml in Fi/TB and 60.5 pg/ml in STR/TB), IL-2 (GM of 27.6 pg/ml in TB alone vs. 11.7 pg/ml in FIL/TB and 13.7 pg/ml in STR/TB) and TNF-α (GM of 1017 pg/ml in TB alone vs. 493.1 pg/ml in FIL/TB and 202.7 pg/ml in STR/TB) as well as Th17 - associated cytokines - IL-17A (GM of 219.2 pg/ml in TB alone vs. 84.1 pg/ml in FIL/TB and 90.6 pg/ml in STR/TB) and IL-17F (GM of 110.2 pg/ml in TB alone vs. 59.9 pg/ml in FIL/TB and 73.6 pg/ml in STR/TB) but not IL-22 in co-infected individuals compared to TB infected individuals. In contrast, we observed significantly higher plasma levels of IL-10 (GM of 116.9 pg/ml in TB alone vs. 209.7 pg/ml in FIL/TB and 177.7 pg/ml in STR/TB) but not TGFβ (data not shown) in helminth co-infected individuals compared to TB-infected individuals. Thus, both helminth infections are associated with profound alterations systemic levels of Th1 and Th17 cytokines in co-infected individuals.

### IL-10 modulates the frequencies of mono- and multifunctional CD4^+^ Th1 cells in filarial-TB co-infection

**Figure 5:**
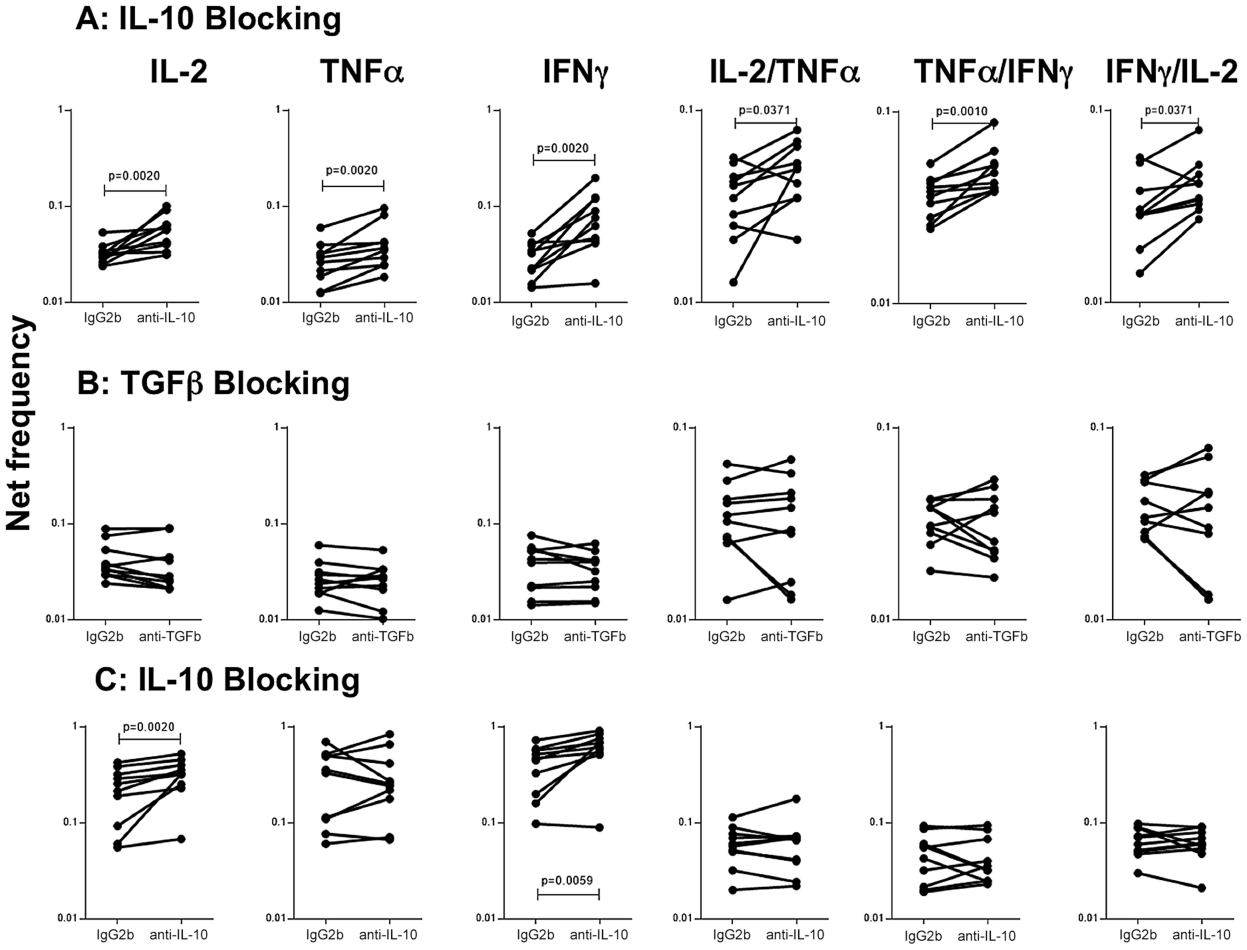
IL-10 but not TGFβ regulates the frequency of CD4^+^ Th1 cells in active TB with concomitant filarial infection. (A) The frequency of mono - and multi - functional CD4^+^ T cells expressing IL-2, TNF-α and IFN-γ following stimulation with CFP-10 and IL-10 neutralization (with anti-IL-10 antibody) in individuals with active TB and concomitant filarial infection (n = 10). (B) The frequency of mono- and multi-functional CD4^+^ T cells expressing IL-2, TNF-α and IFN-γ following stimulation with CFP-10 and TGFβ neutralization (with anti- TGFβ antibody) in individuals with active TB and concomitant filarial infection (n = 10). (C) The frequency of mono- and multi-functional CD4^+^ T cells expressing IL-2, TNF-α and IFN-γ following stimulation with CFP-10 and IL-10neutralization (with anti-IL-10 antibody) in individuals with active TB and no concomitant helminth infection (n = 10). Antigen – stimulated frequencies are shown as net frequencies with the baseline levels subtracted. Each line represents a single individual. P values were calculated using the Wilcoxon signed rank test.

To determine the role of IL-10 and other known immunomodulatory cytokines (e.g.TGFβ in the modulation of CD4^+^ Th1 cells in active TB with concomitant helminth infection, we measured the frequency of cells following stimulation with the TB antigen -CFP-10 in the presence or absence of anti-IL-10 or anti-TGFβ neutralizing antibody in FIL/TB and TB alone individuals (n = 10). As shown in Figure 5A, IL-10 neutralization resulted in significantly increased frequencies of monofunctional (IL-2 or INF-γ or TNF-α expressing) and multifunctional (IL-2/IFN-γ or IFN-γ/TNF-α or IL-2/TNF-α co-expressing) Th1 cells in FIL/TB individuals. In marked contrast, as shown in Figure 5B, TGFβ neutralization had no significant effect on the frequencies of mono- or multi - functional Th1 cells. On the other hand, IL-10 neutralization resulted in significantly increased frequencies of monofunctional (IL-2 or INF-γ or TNF-α expressing) but not multifunctional (IL-2/IFN-γ or IFN-γ/TNF-α or IL-2/TNF-α co-expressing) Th1 cells in TB alone infected individuals (Figure 5C). Thus, IL-10 plays an important role in the modulation of CD4^+^ Th1 cells in FIL/TB co-infection.

## Discussion

Helminth infections afflict over 1.5 billion people worldwide, while Mtb infects one third of the world's population resulting in a million deaths per year [6]. The overlapping geographic distributions of the helminth infections and tuberculosis demonstrate very clearly that, on a population level, the potential for interaction among these various pathogens can occur. A wide variety of studies have been performed to examine the possible effect of helminth infection on the induction of a protective immune response against mycobacteria [22], [23]. Both intestinal and systemic helminths have been shown to modulate proliferation and IFN-γ production in response to PPD in helminth – latent TB coinfected individuals [22], [23]. Some of these effects have been shown to be reversible following anthelmintic chemotherapy [22]. Indeed, we have previously demonstrated that concurrent filarial infection could inhibit the generation of potentially protective Th1 and Th17 immune responses in latent TB infected individuals [8]. In addition, we have also shown that concomitant hookworm infection modulates the frequency of Th1 and Th17 cytokine-producing cells in latent TB [24]. The immunogenicity of BCG vaccination has been shown to be impaired in helminth-infected individuals, and this is associated with enhanced TGF-β production but not enhanced Th2 responses [25], while there exists an inverse association between BCG immunization and intestinal nematode infection [26]. Despite these studies on the interaction of helminth infection and latent TB or TB vaccination, the relationship of helminth infection on the development of active tuberculosis or outcome following treatment is not completely clear.

The two major subsets of CD4^+^ T cells that form an important component of adaptive immune responses to TB are Th1 and Th17 cells [11], [12]. Th1 responses are known to be important in resistance to TB, while Th17 responses are known to be important in inducing and maintaining memory and recall responses to TB [12]. Finally, multifunctional Th1 cells are also thought to play an important role in protection against TB disease [27]. Because immune-mediated protection against Mtb is characterized by strong mycobacterium-specific Th1 and Th17 responses [12], it has been postulated that coincident infections with helminth parasites could modulate these immune responses by driving Th2 and/or Tregs that induce anti-inflammatory responses [6]. Therefore, we have examined the effect of helminth infection on TB - antigen specific immune responses in individuals with active microbiologically confirmed pulmonary TB.

Our data reveal significant alterations in the baseline frequencies of mono - and multifunctional CD4^+^ and CD8^+^ Th1 and Th17 cells in TB-infected individuals with active helminth infection. This is associated with perturbations in the homeostatic or steady - state levels of Th1 and Th17 cytokines in pulmonary TB individuals in comparison to co-infected individuals as well. Our examination of plasma levels of these cytokines clearly reveals that a profound depression of both Th1 and Th17 cytokines is found in those with helminth infection and active TB. Amongst all the cytokines, IFN-γ and TNF-α are known to be critically responsible for protection against TB [11]. Therefore, the diminished circulating levels of these cytokines in helminth co-infected individuals, suggest an impairment in Th1 responses in pulmonary TB with coincident filarial infection. In addition, the diminished systemic production of IL-2, IL-17A and IL-17F also indicate a more extensive impairment in Th1 and Th17 responses in co-infection settings. Thus, helminth infection appears to be associated with homeostatic alterations in the Th1 and Th17 cellular responses in pulmonary TB.

Our study highlights the association of filarial co-infection with a profound impairment in TB - antigen specific CD4^+^ Th1 and Th17 responses. Our data on STR/TB co-infection also reveals remarkably similar yet more pronounced effects of helminth infection on CD4^+^ T cell responses in active TB. Thus, co-infected individuals exhibit a spontaneous deficiency in the frequencies of Th1 and Th17 cells and a much more potent deficiency in the expansion of mono - and multifunctional Th1 and Th17 cells in response to Mtb-specific antigens. In contrast, our data suggest that the intrinsic potential of CD4^+^ T cells to respond to polyclonal stimulation and induce Th1 and Th17 cytokine expression is unaltered in the presence of coincident helminth infection. CD4^+^ T cells expressing IL-2 alone or those co-expressing IL-2 and IFN-γ or TNF-α and IFN-γ have been show to be potential correlates of protective immunity to Mtb [18], [28]. Similarly, multifunctional CD4^+^ T cells co-expressing IFN-γ, TNF-α and IL-2 have also been shown to correlate with immunity to Mtb in a study comparing smear-positive TB to those with smear-negative TB or latent TB [16]. Thus, mono – and multifunctional Th1 cells clearly play an important role in susceptibility or resistance to infection and/or disease. In addition, Th17 cells, secreting one or more cytokines, are also known to play an important role in protective memory responses in TB infection [29]. Since multifunctional T cell responses are known to be better correlates of protective immunity and also to be more persistent [30], the impairment of multifunctional CD4^+^ Th1 and Th17 cells could potentially have an impact on the clinical course of TB disease in co-infected individuals.

Although a role for CD4^+^ T cells in protection against Mtb is well established, there is also a large body of evidence derived from both humans and animal models that suggest an essential role for CD8^+^ T cells [12], [21] as well. CD8^+^ T cells are known producers of Th1 and Th17 associated cytokines and also possess direct antimicrobial activity through granule-exocytosis dependent mechanisms [12]. Since helminth infections can also modulate CD8^+^ T cell responses, we examined the effect of co-incidental helminth infection on CD8^+^ T cell cytokine responses in active TB. Similar to the effect on CD4^+^ T cells, helminth infections appear to exhibit a profound inhibitory effect on the expression of Th1 and Th17 associated cytokines in the context of pulmonary TB. Alterations in cytokine producing CD4^+^ and CD8^+^ T cell subsets could be the result of altered T cell numbers at baseline. Our data suggest that helminth infections are not associated with any such alterations (see Table 1). Moreover, our data also clearly reveal that helminth infections have very little effect on the naive and memory compartmentalization of CD4^+^ and CD8^+^ T cells in active TB. In addition, while the induction of natural Tregs by filarial infections is a major mechanism by which these infections could dampen host immune responses [31], our data also clearly indicate no significant difference in the frequency of nTregs between helminth - infected and uninfected individuals, suggesting that nTreg expansion might not play an important role in modulation of the T cell subsets observed in the present study.

The other major mechanism by which helminth infections are known to alter immune responses to bystander antigens is by the production of immuno-modulatory cytokines - IL-10 and TGFβ [3]. Indeed, filarial infections are known to be associated with an IL-10 dominant cytokine milieu [31]. Moreover, helminth infections were associated with elevated circulating levels of IL-10 in the co-infected individuals, implicating a potential regulatory role for IL-10 in co-infections. Our data on the role of IL-10 and TGFβ in the helminth infection associated modulation of CD4^+^ Th1 responses implicate IL-10 as the major player in the down modulation of Th1 responses in active TB, at least in the context of filarial infections. Moreover, our data also reveal an important role for IL-10 in the down regulation of both mono - and multifunctional Th1 cells in this setting. Interestingly, TGFβ appeared to have a negligible effect on the modulation of the Th1 response to TB antigen, although an effect on Th17 responses or CD8^+^ T cell responses cannot be excluded. In addition, while IL-10 also appears to play an important role in down modulation of Th1 responses in active TB individuals without helminth infection, this effect appears to be selective to mono- functional Th1 cells only. In contrast, filarial infection modulated effector CD4+ T cell responses encompass both mono- and multi-functional Th1 cells. Our data, therefore, suggest a major role for IL-10 in the regulation of immune responses of active TB.

Our findings suggest that in the presence of coincident helminth infection, the ability to restore homeostatic CD4^+^ and CD8^+^ T cell responses in active disease could be worsened. Our study did not have the sample size required to assess the impact of helminth infection on severity of disease or bacterial burdens but the immunological correlates nevertheless highlight a potentially deleterious effect of filarial infection on active TB. In addition, the major strength of our study is the finding that two different helminth infections, with different modes of transmission as well as localization, are both associated with down modulation systemic and antigen - specific immune responses in active TB. Our findings, therefore, have significant implications for treatment and vaccine discovery in TB and suggest that treatment of concomitant helminth infections could have an impact on both the clinical course of TB as well as on vaccine studies in TB-endemic areas.

## Materials and Methods

### Ethics statement

All individuals were examined as part of a clinical research protocol approved by Institutional Review Board of the National Institute for Research in Tuberculosis, and informed written consent was obtained from all participants.

### Study population

We studied a group of 50 individuals with active pulmonary TB, 17 of whom were infected with *W. bancrofti* (hereafter FIL/TB) and 13 of whom had *S. stercoralis* (hereafter STR/TB) infection in Tamil Nadu, South India (Table 1). Another set of 10 individuals with active pulmonary TB and coincident filarial infection and 10 individuals with active TB alone were used for cytokine neutralization experiments. Active pulmonary TB was diagnosed microbiologically on the basis of being at least culture positive for Mtb by solid cultures in LJ medium (some were also sputum smear positive). Filarial infection was diagnosed by the presence of circulating filarial antigen by the TropBio Og4C3 enzyme-linked immunosorbent assay (ELISA) (Trop Bio Pty. Ltd, Townsville, Queensland, Australia). Strongyloides infection was diagnosed by the presence of IgG antibodies to the 31-kDa recombinant NIE antigen by the Luciferase Immunoprecipitation System Assay, as described previously [32]. All individuals were HIV negative and anti-tuberculous and anthelmintic treatment naive. The two groups of individuals did not differ significantly in the radiological extent of disease or bacillary burden (as estimated by smear grades).

### Hematological parameters

Leukocyte counts and differentials were performed on all individuals using the Act-5 Diff hematology analyzer (Beckman Coulter).

### Flow cytometry analysis

Flow cytometry acquisition was done on BD FACS Canto II (BD Biosciences, San José, CA, USA). Analysis was done using FlowJo software v9.4.10 (TreeStar Inc., Ashland, OR, USA).

### Total T cells and naïve, memory, and regulatory T cell subsets

Absolute CD4^+^T cell counts were enumerated in whole blood using BD Multiset 6-Color TBNK cocktail (BD Biosciences). Naïve and memory T cell phenotyping was performed using FITC-CD45RA (BD Pharmingen, BD Biosciences) and APC-CCR7 (eBioscience, San Diego, CA, USA) staining in CD4^+^ and CD8^+^ T cells. Naïve cells were classified as CD45RA^+^CCR7^+^, effector memory cells as CD45RA^−^CCR7^−^, and central memory cells as CD45RA^−^CCR7^+^. Natural Tregs (nTregs) were classified as CD4^+^CD25^+^Foxp3^+^CD127^dim^ (BD Pharmingen and eBioscience).

### Antigens

Mycobacterial antigens— recombinant early secreted antigen-6 (ESAT-6) and culture filtrate protein-10 (CFP-10) (Fitzgerald Industries Intl. Inc, Acton, MA)— were used as the antigenic stimuli. These antigens contain epitopes reactive to both CD4^+^ and CD8^+^ T cells [33]. Final concentrations were 10 µg/ml for ESAT-6 and CFP-10. Anti-CD3 at a concentration of 10 µg/ml was used as the positive control stimuli.

### *In vitro* culture

In vitro cultures and subsequent intracellular cytokine staining was performed. Whole blood cell cultures were performed to determine the intracellular levels of cytokines. Briefly, whole blood was diluted 1∶1 with RPMI-1640 medium supplemented with penicillin/streptomycin (100 U/100 mg/ml), L-glutamine (2 mM), and HEPES (10 mM) (all from Invitrogen, San Diego, CA) and distributed in 12-well tissue culture plates (Costar, Corning Inc., Corning, NY). The cultures were then stimulated with ESAT-6 or CFP-10 or anti-CD3 or media alone in the presence of the costimulatory molecules CD49d/CD28 at 37°C for 6 h. FastImmune Brefeldin A solution (10 µg/ml) was added after 2 h. After 6 h, centrifugation, washing, and red blood cell lysis were performed. Cells were fixed using cytofix/cytoperm buffer (BD Biosciences) and cryopreserved at −80°C. For cytokine neutralization experiments, whole blood from individuals with filariasis and active TB or active TB alone (n = 10) was cultured in the presence of anti-IL-10 (5 µg/ml) or anti-TGFβ (5 µg/ml) or isotype control antibody (5 µg/ml) (R& D Sytems) for 6 h following which CFP-10 and brefeldin A was added and cultured for a further 12 h.

### Intracellular cytokine staining

The cryopreserved cells were thawed, washed, and then stained with surface antibodies for 30–60 min. Surface antibodies used were CD3 (Amcyan), CD4 (APC-H7), and CD8 (PE-Cy7). The cells were washed and permeabilized with BD Perm/Wash buffer (BD Biosciences) and stained with intracellular cytokines for an additional 30 min before washing and acquisition. Cytokine antibodies used were IFN-γ, TNF-α, IL-2, IL-17A, IL-17F, and IL-22. Eight-color flow cytometry was performed on a FACSCanto II flow cytometer with FACSDiva software v.6 (Becton Dickinson and Company, Cockeysville, MD). Lymphocyte gating was set by forward and side scatter, and 100,000 lymphocyte events were acquired. Gating for CD4^+^ T cells expressing cytokines was determined by FMO (fluorescence minus one). Data were collected and analyzed using Flow Jo software (TreeStar Inc., Ashland, OR). All data are depicted as frequency of CD4^+^ T cells expressing cytokine(s). Baseline values following media stimulation are depicted as baseline frequency, while frequencies following stimulation with antigens or PMA/ionomycin are depicted as net frequencies (with baseline values subtracted).

### Immunoassays

Plasma cytokines on all 50 individuals were measured using Bioplex multiplex cytokine assay system (Biorad). The cytokines analyzed were IL-2, IFN-γ, TNF-α, IL-10, IL-17A, IL-17F and IL-22. TGFβ levels were measured using a standard ELISA kit from R&D Systems.

### Statistical analysis

Data analyses were performed using GraphPad PRISM (GraphPad Software, Inc., San Diego, CA, USA). Geometric means (GM) were used for measurements of central tendency. Comparisons were made using either the Kruskal-Wallis test with Dunn's multiple comparisons (unpaired comparisons) or the Wilcoxon signed rank test (paired comparisons).
